# Altruism of Shiga toxin-producing *Escherichia coli*: recent hypothesis versus experimental results

**DOI:** 10.3389/fcimb.2012.00166

**Published:** 2013-01-04

**Authors:** Joanna M. Łoś, Marcin Łoś, Alicja Węgrzyn, Grzegorz Węgrzyn

**Affiliations:** ^1^Laboratory of Molecular Genetics, Department of Molecular Biology, University of GdańskGdańsk, Poland; ^2^Institute of Physical Chemistry, Polish Academy of SciencesWarsaw, Poland; ^3^Phage ConsultantsGdańsk, Poland; ^4^Department of Microbiology, University of SzczecinSzczecin, Poland

**Keywords:** enterohermorrhagic *Escherichia coli*, Shiga toxin, lambdoid bacteriophages, prophage induction, bacterial altruism

## Abstract

Shiga toxin-producing *Escherichia coli* (STEC) may cause bloody diarrhea and hemorrhagic colitis (HC), with subsequent systemic disease. Since genes coding for Shiga toxins (*stx* genes) are located on lambdoid prophages, their effective production occurs only after prophage induction. Such induction and subsequent lytic development of Shiga toxin-converting bacteriophages results not only in production of toxic proteins, but also in the lysis (and thus, the death) of the host cell. Therefore, one may ask the question: what is the benefit for bacteria to produce the toxin if they die due to phage production and subsequent cell lysis? Recently, a hypothesis was proposed (simultaneously but independently by two research groups) that STEC may benefit from Shiga toxin production as a result of toxin-dependent killing of eukaryotic cells such as unicellular predators or human leukocytes. This hypothesis could make sense only if we assume that prophage induction (and production of the toxin) occurs only in a small fraction of bacterial cells, thus, a few members of the population are sacrificed for the benefit of the rest, providing an example of “bacterial altruism.” However, various reports indicating that the frequency of spontaneous induction of Shiga toxin-converting prophages is higher than that of other lambdoid prophages might seem to contradict the for-mentioned model. On the other hand, analysis of recently published results, discussed here, indicated that the efficiency of prophage excision under conditions that may likely occur in the natural habitat of STEC is sufficiently low to ensure survival of a large fraction of the bacterial host. A molecular mechanism by which partial prophage induction may occur is proposed. We conclude that the published data supports the proposed model of bacterial “altruism” where prophage induction occurs at a low enough frequency to render toxin production a positive selective force on the general STEC population.

## Background

*Escherichia coli* is a Gram-negative bacterium, commonly known as an intestinal commensal of mammals including humans. However, some *E. coli* strains are pathogenic to humans. These include the Shiga toxin-producing *E. coli* (STEC), particularly a subset of strains classified as enterohemorrhagic *E. coli* (EHEC). The development of severe diseases such as the hemolytic uremic syndrome (HUS) and hemorrhagic colitis (HC) by these strains, including the most intensively studied serotype O157:H7, depends on production of Shiga toxins (Hunt, 2010).

HUS and HC are possible complications of infection of humans by EHEC (Gyles, 2007). Children and elderly people are the groups at highest risk for HUS. Among patients infected with EHEC and suffering from diarrhea, 3–15% develops HUS (Razzaq, 2006). This syndrome is characterized by acute renal failure, hemolytic anemia, and thrombocytopenia. Other organs such as the lung, pancreas, and heart may also be damaged. Moreover, some patients suffer additionally from nervous system dysfunctions, which may include lethargy or disorientation (Obata et al., 2008 and references therein). Mortality of patients with STEC-associated HUS has been estimated at up to 10% (Razzaq, 2006; Gyles, 2007).

The severity of EHEC-associated disease can be exemplified by the recent outbreak that occurred in Germany in 2011, when out of about 4000 symptomatic infections, 50 patients died (Mellmann et al., 2011; Beutin and Martin, 2012; Bloch et al., 2012; Karch et al., 2012; Werber et al., 2012). In fact, the strain which caused that outbreak was of the O104:H4 serotype and produced a combination of virulence factors characteristic of enteroaggregative *E. coli* (EAEC) and EHEC (reviewed by Karch et al., 2012). It appears that this strain was EAEC which acquired *stx* genes by lysogenization with a Shiga toxin-converting bacteriophage (Laing et al., 2012). The specific combination of enhanced adhesion, survival fitness, antibiotic resistance, and Shiga toxin production may explain the high virulence of this particular strain (Karch et al., 2012). This also underlines the importance of Shiga toxins to the virulence of *E. coli* strains, which would otherwise be significantly less dangerous to humans.

Shiga toxins are proteins consisting of two types of subunits, A and B, forming a heterohexamere composed of a single A-subunit and five B-subunits (AB_5_). The C-terminal part of the A-subunit is anchored to the B-subunit pentamer (Law, 2000). B-subunits recognize a specific cell-surface receptor (Gb3) on eukaryotic cells. The toxin enters Gb3 expressing cells by receptor-mediated endocytosis, following which it is translocated via the endosome to the lysosome for degradation, or it undergoes retrograde transport from the early endosome through the Golgi-apparatus and to the endoplasmic reticulum. In the latter case, there is an initial proteolytic cleavage of the A-subunit, resulting in formation of an A1 fragment connected to an A2 fragment by a disulfide bond. The A2 fragment remains associated with the B pentamer, and this complex is released from A1 following reduction of the disulfide bond (which probably occurs in the endoplasmic reticulum). Thus, the A1 polypeptide is translocated from the ER to the cytoplasm (for a detailed description of the retrotranslocation and processing of Shiga toxin, see the following references: Garred et al., 1995a,b, 1997; LaPointe et al., 2005; Yu and Haslam, 2005; Tam and Lingwood, 2007). In the cytoplasm, this toxin acts as an N-glycosidase that depurinates the sarcin/ricin loop of a single adenine residue in the 28S rRNA (Obrig et al., 1987; Endo et al., 1988). This causes an inhibition of binding of amino-acyl-tRNA to the ribosome and cessation of protein synthesis, leading to cell death (for a review, see Law, 2000).

Contrary to many other virulence factors, genes encoding Shiga toxins (*stx* genes) are not of *E. coli* chromosome origin, but are part of lambdoid prophages (Allison, 2007). In a majority of STEC strains analyzed to date, the *stx* genes are under control of the late phage promoter, pR′ (Wagner et al., 2001a, 2002). In lambdoid phages, the expression of most genes (except for *c*I in all lambdoid phages, and a few genes in other phages) is strongly inhibited in the prophage state, due to the activity of the cI repressor (Ptashne, 2004; Węgrzyn and Węgrzyn, 2005; Riley et al., 2012). Thus, in bacteria lysogenic for Shiga toxin-converting bacteriophages, expression of the *stx* genes is repressed. This implies that production of Shiga toxins must be preceded by prophage induction, which has been demonstrated by Herold et al. (2004) and Waldor and Friedman (2005). It is worth reminding that the most efficient induction of lambdoid prophages occurs under conditions that induce the bacterial SOS response (a response to DNA damage or DNA synthesis perturbation) and is dependent on the host-encoded RecA protein (Ptashne, 2004; Węgrzyn et al., 2012).

The regulatory mechanism described above raised an intriguing question. Namely, induction of a lambdoid prophage leads irreversibly to bacteriophage lytic development, which ends with production of progeny virions and their liberation after host cell lysis (Ptashne, 2004; Węgrzyn and Węgrzyn, 2005). Actually, lysis allows for the effective release of Shiga toxins. However, this means that a bacterial cell producing Shiga toxin must die before large quantities of the toxin can act as a virulence factor. Therefore, one might ask what is the benefit for a bacterium to produce Shiga toxin if its death is strongly coupled to *stx* expression? If there is no such benefit, one might predict a selective pressure against toxin-producing bacteria, resulting in accumulation of mutations in *stx* genes and eventual elimination of STEC from the environment. However, this is not the case, and therefore it would be expected that *E. coli* should incur a benefit from lysogeny by Shiga toxin-converting phages. Recently, two different groups proposed (simultaneously but independently) a hypothesis explaining how STEC might benefit from Shiga toxin production (Łoś et al., 2011; Mauro and Koudelka, 2011). The hypothesis is that Shiga toxin protects STEC from attack by unicellular predators and possibly neutrophils (although Shiga toxin has not been shown to be directly toxic to neutrophils, its production might be a response to neutrophil-mediated attack). This hypothesis, while perhaps intriguing, might also be recognized as controversial, as it could be difficult to prove it in the laboratory. Nevertheless, if true, the hypothesis should be compatible with existing experimental data. Therefore, in our opinion, it requires an assessment of probability in light of published results.

## Is virulence of STEC to humans coincidental?

Although STEC strains produce virulence factors and cause severe symptoms in infected humans, they might be assessed as non-classical human pathogens. This is because human-to-human transmission of STEC is relatively rare outside of an outbreak situation and therefore is probably insufficient to sustain populations of these bacteria (Brandl, 2006; Vaillant et al., 2009; Aldabe et al., 2011; Locking et al., 2011; Rotariu et al., 2012). In fact, cattle and other ungulates, to which these bacteria are usually non-pathogenic, are considered natural hosts for STEC (Dean-Nystrom et al., 1988; Hancock et al., 1988). Therefore, Brandl (2006) has proposed that STEC virulence in humans is coincidental with the biological role for Shiga toxin being unrelated to human infection. According to this hypothesis, synthesis of Shiga toxins by *E. coli* may enhance survival of bacteria in food vacuoles of protozoan predators. In fact, results of subsequent experimental studies indicate that the presence of Shiga toxin-converting prophages augment the fitness of *E. coli* in the presence, but not the absence, of a protozoan predator, *Tetrahymena pyriformis* (Steinberg and Levin, 2007). Moreover, the carriage of the *stx* gene on a prophage increased the rate of bacterial survival in the food vacuoles of this ciliate (Steinberg and Levin, 2007).

Similar studies additionally support a role for *stx* genes in the survival of *E. coli* outside the human intestine. It was demonstrated that another bacterivorous, protozoan predator, *T. thermophila*, was killed when co-cultured with bacteria lysogenic with Shiga toxin-converting bacteriophage (Lainhart et al., 2009). Interestingly, this eukaryotic unicellular predator died in the presence of purified Shiga toxin, strongly suggesting that this toxin may be used as an anti-predator agent by bacteria. It is possible that the mechanism by which Shiga toxin kills *Tetrahymena* is analogous to that resulting in toxicity to human cells.

Another interesting observation was that in the presence of catalase, an enzyme that breakdowns hydrogen peroxide, STEC-dependent *Tetrahymena* killing in co-cultures diminished (Lainhart et al., 2009). *Tetrahymena* is known to produce H_2_O_2_ (Fok and Allen, 1975), which may be used to damage bacterial cells during attack by this predator, however, one might speculate that if Shiga toxin-converting prophages are induced by hydrogen peroxide, this strategy is not effective. In fact, *recA* mutations in *E. coli*, which prevented the bacterial SOS response [a process required to trigger lambdoid prophage induction (Ptashne, 2004; Węgrzyn and Węgrzyn, 2005)], protected *Tetrahymena* from being killed in co-cultures with STEC (Lainhart et al., 2009). Moreover, induction of lambdoid prophages was demonstrated experimentally in hydrogen peroxide-treated lysogenic bacteria (Łoś et al., 2009, 2010).

## The hypothesis

**Figure 1:**
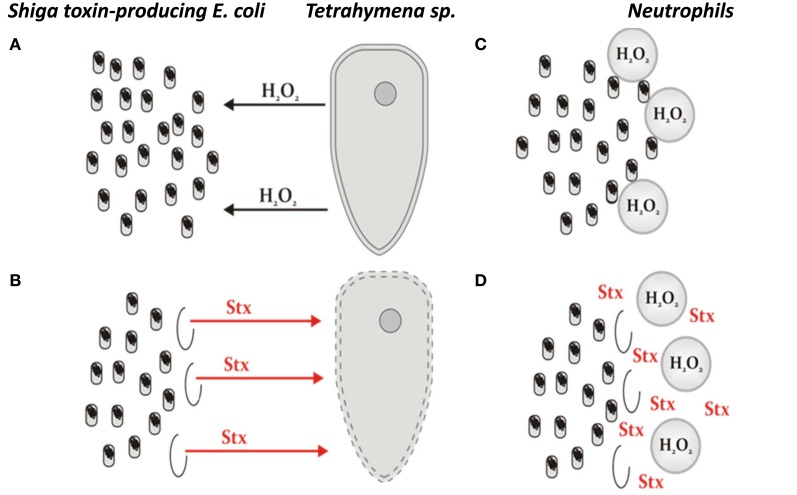
**The model of STEC altruism, representing the hypothesis on the benefit which *E. coli* lysogenic with Shiga toxin-converting phages may gain from production of Shiga toxins coupled with prophage induction and subsequent cell death. (A)** A protozoan predator (exemplified by *Tetrahymena*) releases hydrogen peroxide to damage bacterial cells. **(B)** In the case of STEC, hydrogen peroxide causes induction of Shiga toxin-converting prophage in a small fraction of bacterial cells, which is, nevertheless, sufficient to produce Shiga toxins (Stx) in amounts enough to kill the predator. **(C)** Neutrophils employ a strategy similar to that used by protozoan predators if human intestine is infected with bacteria recognized as aliens. **(D)** The response of STEC to neutrophils' attack is analogous to that employed by this bacterium to faith against unicellular eukaryotic predators. The original hypothesis was presented in two articles (Łoś et al., 2011; Mauro and Koudelka, 2011).

The results summarized in the preceding subsection suggest that production of hydrogen peroxide by eukaryotic unicellular predators, like *Tetrahymena*, may induce Shiga toxin-converting prophages in STEC, resulting in production and release of Shiga toxin. These data provided a basis for the hypothesis that under such conditions, the predator would be killed, which might be beneficial for the bacteria (Łoś et al., 2011; Mauro and Koudelka, 2011). If we consider that infection of humans by STEC is coincidental, one might ask why these bacteria produce Shiga toxins in the intestine? As suggested by the authors of this bacterial “altruism” hypothesis, the occurrence of hydrogen peroxide in the human intestine during infection is possible since human neutrophils may produce H_2_O_2_ in response to STEC (Wagner et al., 2001b), a strategy similar to that used by *Tetrahymena* to hunt bacteria. Moreover, it was demonstrated that bacteria present in the human intestine can cause the generation of reactive oxygen species (Kumar et al., 2007). Therefore, the hypothesis can be presented schematically as shown in Figure 1. However, since prophage induction leads to lytic development and killing of the host cell, the problem with the hypothesis is that it is difficult to imagine how already dead bacteria may benefit from killing a predator or neutrophils (or other human cells). Therefore, the bacterial “altruism” hypothesis presented here (Figure 1) requires that prophage induction only occur in a small fraction of the STEC population. So, what is the experimental evidence rejecting or supporting this model?

### Arguments against the hypothesis

A necessary condition of the hypothesis, i.e., that there can be minimal STEC prophage induction, might appear unlikely in the light of certain published data. Namely, it was demonstrated that in *E. coli*, the spontaneous induction (i.e., without treatment of lysogenic cells by any specific induction agent) of Shiga toxin-converting prophages is significantly more frequent than that of other known lambdoid prophages (Livny and Friedman, 2004; Shimizu et al., 2009). Moreover, specific conditions, such as high hydrostatic pressure, can induce prophages carrying Shiga toxin genes (Aertsen et al., 2005). As demonstrated recently, these prophages can also be induced in a RecA-independent manner with the chelating agents, EDTA or citrate, and various chelating compounds may occur in the human intestine (Imamovic and Muniesa, 2012). Furthermore, unlike *E. coli* lysogenic with λ and some other lambdoid phages, STEC are known to often harbor more than one Shiga toxin-converting prophage, and double lysogens were recently demonstrated to be more sensitive to inducing agents than single lysogens (Fogg et al., 2012). This suggests that maintenance of a stable prophage is less likely when multiple phage genomes are inserted into the *E. coli* chromosome. These results indicate that in the absence of specific agents promoting prophage induction, Shiga toxin-converting prophages are less stable in *E. coli* hosts than other lambdoid prophages. Moreover, additional mechanisms of RecA-independent prophage induction could exist in phages bearing Shiga toxin genes. Therefore, the hypothesis under debate might appear doubtful.

Some STEC strains, including *E. coli* O157, are able to survive and replicate inside protozoal cells, specifically the common protozoan *Acanthamoeba polyphaga* (Barker et al., 1999), and another protozoan species, *A. castellani*, have been demonstrated to be hosts for *E. coli* O157:H7 (Carruthers et al., 2010). These observations corroborate data presented in previous publications, which suggested that protozoa can be hosts for bacteria, including those pathogenic to humans (Barker and Brown, 1994; Lloyd et al., 1996). Therefore, one might assume that growth of pathogenic bacteria inside protozoan cells could enhance the environmental survival of these prokaryotes. Accordingly, it has been proposed that the residing of pathogenic bacteria within protozoa may increase bacterial virulence and resistance to antibiotics (Barker and Brown, 1994; Lloyd et al., 1996). On the other hand, when studying interactions between rumen ciliates and STEC, Burow et al. (2005) failed to detect STEC-specific DNA in protozoal fractions after fractionation of the co-cultures. They concluded that ruminal protozoa are unlikely to be a major factor in the survival of STEC in ruminants. Nevertheless, they also concluded that these ciliates are neither hosts nor predators to STEC (Burow et al., 2005). These data suggest that if ruminal mammals are natural hosts for STEC, then the ciliates present are not likely predators of STEC.

### Arguments for the hypothesis

Although Shiga toxin-converting prophages undergo spontaneous induction more frequently than other lambdoid phages (see previous subsection), the actual frequency of the switch from lysogenic to lytic development in an untreated bacterial host is still low (Livny and Friedman, 2004; see also Łoś et al., 2011). It was estimated that spontaneous induction occurs in approximately 0.005% of cells per generation in bacteria lysogenic with the Shiga toxin-converting phage H-19B (Livny and Friedman, 2004). Therefore, in light of the discussed hypothesis, it is important to consider what fraction of lysogenic cells is subjected to prophage induction under the specific conditions resulting in enhanced Shiga toxin production.

It was found that among the many tested factors and conditions that are potentially present in the human intestine during a bacterial infection, hydrogen peroxide is a potent stimulator of lambdoid prophage induction (Łoś et al., 2009). However, comparison of the efficiency of prophage induction after treatment of lysogenic bacteria with either H_2_O_2_ or mitomycin C (an antibiotic employed in laboratories as an efficient SOS inducer when administered at relatively high concentrations) revealed that H_2_O_2_ is significantly less effective in both laboratory *E. coli* strains (Łoś et al., 2009) and a natural isolate of STEC (Łoś et al., 2010). Based on these results, under optimal prophage inducing concentrations of H_2_O_2_ (3 mM), induction occurs at most in a small percentage of cells (the highest calculated value was 1.6%, Table 1). In contrast to that of H_2_O_2_, treatment of bacteria with mitomycin C (1 μg/ml) induces lytic development in about 10–30% of lysogenic cells in the culture (Table 1). In fact, these values are still significantly higher than those calculated for bacteriophage λ (Table 1), corroborating the conclusions from previous reports on lower stability of Shiga toxin-converting prophages. Nonetheless, during treatment of STEC with hydrogen peroxide only a small fraction of bacterial population is killed due to prophage induction, which is still sufficient to produce relatively large amounts of Shiga toxin due to intensive replication of phage DNA and transcription from phage promoters during lytic development (Nejman et al., 2009, 2011; Nejman-Faleńczyk et al., 2012).

**Table 1:** **Efficiency of prophage induction after treatment of *E. coli* lysogenic cells with various compounds**.

**Bacteriophage^a^**	**Calculated fraction of induced lysogenic cells**	
	**3% H_2_O_2_**	**1 μg/ml mitomycin C**
λ	0.03%	1.5%
Φ24_B_	0.3%	11%
933W	0.03%	17%
P22	1.6%	32%
P27	0.1%	24%
P32	0.2%	28%

The calculation is based on previously reported results (Łoś et al., 2009), and represents an estimated average fraction of cells in which prophage has been induced, assuming equal efficiency of progeny phage formation in every cell.

a Bacteriophages Φ24_B_, 933W, P22, P27, and P32 were originally isolated as Shiga toxin-converting phages from lysogenic STEC strains (for details, see Łoś et al., 2009, and references therein).

Interestingly, when we analyzed other published results, it appeared clear that conditions expected to occur naturally (i.e., not only in laboratories, like high concentration of mitomycin C) can provoke prophage induction at low frequencies. As previously discussed, adding chelating compounds, such as EDTA, to STEC cultures promotes phage induction and results in high titers of Shiga toxin-converting phage (Imamovic and Muniesa, 2012). However, considering that the average phage lytic cycle produce roughly 100 pfu/cell, re-evaluation of the formentioned study (Imamovic and Muniesa, 2012) suggests that prophage induction occurs in at most 1% of cells. This is over an order of magnitude less than in experiments performed with mitomycin C (Imamovic and Muniesa, 2012). Therefore, it appears that the requirement of low efficiency prophage induction in combination with effective toxin production may occur following exposure to naturally occurring agonists of prophage induction.

When considering the survival or even growth of STEC inside cells of protozoan microorganisms (Barker et al., 1999; Carruthers et al., 2010), one should take into consideration that some protozoans might have a commensal/symbiotic interaction with STEC while others may predate on STEC. Thus, while *E. coli* strains may benefit from interactions with *A. polyphaga* and *A. castellani* (hosts), they may be at risk interacting with *T. pyriformis* or *T. thermophila* (predators). Interestingly, Carruthers et al. (2010) found that expression of *stx* genes was enhanced in STEC occurring inside *A. castellani* cells relative to planktonic cultures. This may suggest that a protozoan cell is not necessarily an optimal habitat for STEC, and that there could be various interactions between the eukaryotic host and the bacterium, which may not be beneficial to both organisms.

The conclusions presented by Burow et al. (2005) that ruminal protozoa are neither hosts nor predators for STEC require additional comments. First, the mixed protozoan-bacterial cultures, used in their experiments, contained representatives of only two genera of ciliates: *Entodinium* and *Epidinium*. Although they started the protozoan cultures from faeces of sheep and cattle, no other genera present in the original rumen fluid had established growth in the mixed culture (Burow et al., 2005). Thus, one may speculate that *Entodinium* and *Epidinium* cannot host STEC while some other ruminal protozoans can. Therefore, the conclusion about a lack of STEC hosting by protozoans in the rumen may be valid for *Entodinium* and *Epidinium* but not necessarily for other genera. Furthermore, the lack of interactions between the ciliates mentioned above and STEC, observed by Burow et al. (2005), could be caused by factors other than the absence of specific phenomena in the rumen. For example, these protozoans might be unable to produce hydrogen peroxide, in contrast to *Tetrahymena*, thus they may not induce production of Shiga toxins. Also, *Entodinium* and *Epidinium* could be more resistant to Shiga toxins than other genera of protozoans. Alternatively, the inability of other ruminal protozoa to grow in a mixed culture with STEC could result from these protozoans being killed by Shiga toxins. If these speculations are true, they could provide an alternative explanation to the results reported by Burow et al. (2005) that suggests a lack of major influence by ruminal protozoa on the survival of STEC in ruminants.

Second, the method of STEC detection in the protozoan fraction after fractionation of the mixed culture was based on PCR-mediated amplification of *stx* genes. The authors (Burow et al., 2005) assumed that if STEC occurred inside protozoan cells, it would be possible to detect *stx*-specific signals. However, while this assumption should be true in the case of living bacteria, the digestion of bacteria by the ciliate might result in DNA degradation and therefore no detection by PCR. Therefore, although the conclusion about a lack of effective colonization of *Entodinium* and *Epidinium* by STEC appears true, a lack of predatory interactions between these ciliates and STEC can still be argued, and it is obvious that predatory interactions occur between other protozoans, like *Tetrahymena*, and STEC (Steinberg and Levin, 2007; Lainhart et al., 2009). The remaining question is whether STEC strains are endangered by protozoan predators only outside the mammalian digestive tract. In summary, although it might appear that previous reports on interactions between protozoan and bacterial cells were incompatible with the hypothesis, more detailed analysis of the published results indicated that this is not the case. Therefore, the hypothesis may still be true and compatible with reported observations on various STEC-protozoan relations.

## Are there other benefits to STEC from shiga toxin production?

The hypothesis under debate assumes that the main role for Shiga toxin production is protecting STEC against either protozoan predators or (perhaps incidentally) the mammalian immunological system. However, are there other benefits to bacteria possible?

An interesting proposal has been made by Robinson et al. (2006). They have demonstrated *in vitro* that the *stx2* mutant of a STEC strain was less effective in adherence to epithelial cells than its wild-type counterpart. Moreover, the mutant revealed a lower capacity to colonize mouse intestine. Therefore, it was concluded that Shiga toxin promotes intestinal colonization, possibly also in humans (Robinson et al., 2006). Some earlier results, indicating specific mutations that reduce adherence of STEC to human colonic epithelial cells (Scott et al., 2003), might also corroborate such a conclusion. In contrast to the proposal of Robinson et al. (2006), experiments performed by Sheng et al. (2006) indicated that *E. coli* O157:H7 requires the presence of intimin, Tir and plasmid pO157 to colonize cattle at the terminal rectal mucosa, while the colonization proceeded normally in the absence of the active *stx* genes.

Even if we assume that Shiga toxin helps bacteria colonize the human intestine, there is still the question concerning the benefit of *stx* expression in lieu of cell death following prophage induction. Thus, the hypothesis under debate may still be attractive despite the putative involvement of Shiga toxin in facilitating colonization of the intestine. Interestingly, it was demonstrated that commensal bacteria present in the human intestine can induce the generation of reactive oxygen species (Kumar et al., 2007). If this is the case during the colonization by STEC, the resulting oxidative stress might cause induction of Shiga toxin-converting prophage in a small fraction of bacterial cells, with subsequent production and liberation of significant amounts of the toxin.

## Putative molecular mechanism for low efficiency prophage induction

If the bacterial “altruism” hypothesis under debate is true, it should be possible to propose a putative mechanism for low efficiency lambdoid prophage induction under natural “inductive” conditions, such as H_2_O_2_ exposure. To elaborate a possible molecular model for such regulation, one needs to find either conditions or a specific experimental system in which efficiency of prophage induction provoked by hydrogen peroxide would be similar to that caused by “strong” inductors (like mitomycin C). In fact, studies on bacteriophage λ, a close relative to Shiga toxin-converting phages, demonstrated that induction of the prophage by H_2_O_2_ is over 2 orders of magnitude more efficient in *E. coli oxyR* mutants than in wild-type hosts (Glinkowska et al., 2010).

The OxyR protein (the *oxyR* gene product) is a transcription regulator and a major regulatory factor stimulated during oxidative stress (Chiang and Schellhorn, 2012). This regulator has been demonstrated to interact with λ DNA at the region of the pR promoter, facilitating repression of this promoter by the phage-encoded cI protein, and stimulating activation of the pM promoter (required for the cI repressor production) (Glinkowska et al., 2010). Both repression of pR and activation of pM are necessary for prophage maintenance (Ptashne, 2004; Węgrzyn and Węgrzyn, 2005). Importantly, the presence of a potential OxyR-binding sequence has been identified close to the *c*I transcription start site in genomes of various lambdoid bacteriophages, including Shiga toxin-converting bacteriophages (Glinkowska et al., 2010). Thus, we propose that the host-encoded OxyR transcription regulator may be employed by the phage to keep the efficiency of hydrogen peroxide-dependent prophage induction at a relatively low level. The bacterial “altruism” hypothesis might then be supported further if such regulation were shown to occur in Shiga toxin-converting prophage.

## Concluding remarks

Recently, two research groups proposed independently a hypothesis that can explain the bacterial benefit from producing Shiga toxins, even if production is coupled with prophage induction and subsequent bacterial cell death (Łoś et al., 2011; Mauro and Koudelka, 2011). This hypothesis is depicted in Figure 1. While it appears that experimental data might not be compatible with this hypothesis, a detailed analysis of the published data demonstrates that the facts corroborate the hypothesis rather than disqualify it. Therefore, the “model of STEC altruism” can be described as follows: outside the mammalian intestine, enteric bacteria are endangered by protozoan predators, which produce and release hydrogen peroxide to damage prokaryotic cells, facilitating their predation. Such a strategy is efficient against most bacteria, but STEC strains, bearing prophages with *stx* genes, have developed a defensive strategy based on production of Shiga toxins which can kill eukaryotic cells. Because production of Shiga toxins is strongly coupled to prophage induction and bacterial cell lysis, the efficiency of Shiga toxin-converting prophage induction must be relatively low, so that a large proportion of the bacterial population survive. In fact, H_2_O_2_-mediated induction of these prophages is of low efficiency, perhaps due to the activity of the OxyR regulatory protein or some similar system that enhances prophage maintenance. This low level prophage induction is, however, sufficient to produce Shiga toxins in amounts large enough to neutralize the predator. If STEC enters the human intestine, it may be recognized as an alien organism, and neutrophils may try to kill STEC by producing hydrogen peroxide and other reactive oxygen species, which is a strategy analogous to that used by protozoan predators. The response of STEC to such an action is also analogous to that employed to neutralize a eukaryotic unicellular predator. However, in the intestine, a population of STEC may survive the attack by the human immunological system rather than the attack of a predator. Since the effects of Shiga toxins on human cells could be as serious as those on protozoan organisms, severe symptoms may develop in infected humans. At the same time, a population of Shiga toxin-producing bacteria may not only survive in the human intestine, but may also be shed in excrement during diarrhea. It is worth noting that under these conditions STEC strains are immune to infection by Shiga toxin-converting bacteriophages as all bacteria lysogenic with lambdoid phages are resistant to infection by the same phage (due to a strong repression of phage lytic development by the cI repressor, present in large amounts in lysogenic cells) (for the description of the immunity analysis of Shiga toxin-encoding bacteriophages see Allison et al., 2003; for reviews on other lambdoid phages see Ptashne, 2004; Węgrzyn and Węgrzyn, 2005; Łoś et al., 2011; Węgrzyn et al., 2012). Therefore, the production of progeny virions and their release is not dangerous for the population of STEC, but facilitate either lysogenization of other *E. coli* strains present in the intestine or further propagation of bacteriophages on such strains if lytic development of phages is chosen after infection of sensitive bacteria. The latter scenario could also result in production of larger amounts of Shiga toxin, which would provide an additional benefit for STEC. Although it was demonstrated that there are multiple integrations sites for Shiga toxin-converting phages in *E. coli* genomes, and that superinfection of *E. coli* lysogenized by isogenic phages bearing *stx* genes can occur, such superinfection leads to lysogeny rather than lysis (Fogg et al., 2007, 2011; Serra-Moreno et al., 2007, 2008). Therefore, STEC are not endangered by Shiga toxin-converting phages isogenic to those maintained in these STEC in the form of prophages. Finally, one might assume that this bacterial “altruism” hypothesis can be further tested experimentally by determining the actual fraction of STEC cells in which prophage induction occurs following treatment with hydrogen peroxide (the current calculation is based on the assumption that efficiency of phage progeny production is equal in every cell after excision of the prophage DNA and subsequent phage lytic development), and by comparing the efficiency of toxin production in STEC culture and in mixed culture containing both STEC and other *E. coli* susceptible to Shiga toxin-converting phage infection.

### Conflict of interest statement

The authors declare that the research was conducted in the absence of any commercial or financial relationships that could be construed as a potential conflict of interest.

## Abbreviations

EAEC: enteroaggregative *Escherichia coli*
EHEC: enterohemorrhagic *Escherichia coli*
pfu: plaque forming units
ER: endoplasmic reticulum
STEC: Shiga toxin-producing *Escherichia coli*
HC: hemorrhagic colitis
HUS: hemolytic uremic syndrome.
